# Circular RNA vaccine in disease prevention and treatment

**DOI:** 10.1038/s41392-023-01561-x

**Published:** 2023-09-11

**Authors:** Dun Niu, Yaran Wu, Jiqin Lian

**Affiliations:** 1 Department of Clinical Laboratory Medicine, Southwest Hospital, Army Medical University (Third Military Medical University), 400038 Chongqing, China; 2 Department of Clinical Biochemistry, Army Medical University (Third Military Medical University), 400038 Chongqing, China

**Keywords:** Nucleic-acid therapeutics, Molecular medicine

## Abstract

CircRNAs are a class of single-stranded RNAs with covalently linked head-to-tail topology. In the decades since its initial discovery, their biogenesis, regulation, and function have rapidly disclosed, permitting a better understanding and adoption of them as new tools for medical applications. With the development of biotechnology and molecular medicine, artificial circRNAs have been engineered as a novel class of vaccines for disease treatment and prevention. Unlike the linear mRNA vaccine which applications were limited by its instability, inefficiency, and innate immunogenicity, circRNA vaccine which incorporate internal ribosome entry sites (IRESs) and open reading frame (ORF) provides an improved approach to RNA-based vaccination with safety, stability, simplicity of manufacture, and scalability. However, circRNA vaccines are at an early stage, and their optimization, delivery and applications require further development and evaluation. In this review, we comprehensively describe circRNA vaccine, including their history and superiority. We also summarize and discuss the current methodological research for circRNA vaccine preparation, including their design, synthesis, and purification. Finally, we highlight the delivery options of circRNA vaccine and its potential applications in diseases treatment and prevention. Considering their unique high stability, low immunogenicity, protein/peptide-coding capacity and special closed-loop construction, circRNA vaccine, and circRNA-based therapeutic platforms may have superior application prospects in a broad range of diseases.

## Introduction

Circular RNAs (circRNAs) are characterized by a single-stranded covalent closed-loop structure without a 5’ cap structure and a 3’ polyadenylated (polyA) tail. Owing to high-throughput RNA sequencing technologies, bioinformatics algorithms and experimental techniques, circRNAs have been identified in large numbers and are thought to have a variety of functions in organisms, even to be involved in disease progression.^1–6^ The best-known circRNAs are noncoding RNAs that function as sponges for microRNAs (miRNAs) or proteins.^7,8^ In addition, circRNAs can serve as scaffolds for protein complex formation and as biomarkers for disease diagnosis.^9,10^ In exceptional cases, circRNAs have also been found to act as transcripts to direct protein encoding.^1,6,11^ The progressive discovery of more complex functions of circRNA warrants deeper study and utilization for disease treatment.

Vaccines originate from antiviral immunity and stimulate T-cell-mediated cellular immunity and B-cell/antibody-mediated humoral immunity.^12,13^ Vaccines have helped to decrease the mortality and morbidity of various diseases and are considered to be one of the greatest achievements of public health.^14,15^ Most vaccines rely on either inactivated or live attenuated technologies to stimulate innate, cellular, and humoral immune responses. The main disadvantages of these vaccines are their susceptibility to hypersensitivity and their variable safety and efficacy.^16^ In addition, subunit vaccines are safe but have some defects, including low immunogenicity, adjuvant requirements, and high cost.^17,18^ In contrast, RNA-based vaccines containing antigenic sequences are being developed, and they have received increasing attention from pharmaceutical companies and researchers worldwide.^19,20^ Unlike plasmid deoxyribonucleic acid and viral vectors, which introduce the risk of mutation caused by gene insertion and/or infection, RNA-based vaccines can directly encode antigens after entering the cytoplasm and are nonintegrative, noninfectious, and well-tolerated.^21,22^ However, mRNA vaccines, as first-generation RNA-based vaccines, are not ideally positioned for fast and economical bulk production. Specifically, mRNA vaccines are extremely unstable, both in terms of storage and in vivo delivery.^23–25^ In the worst scenarios, strategies to address this limitation dramatically increase the cost of manufacturing mRNA vaccines.^26^ Accordingly, alternative approaches are desirable for fully unleashing the potential of RNA-based vaccines.

Fortunately, breakthroughs in the in vitro synthesis of circRNA have made the next generation of RNA-based vaccines possible. Starting in 2022, the expression of relevant antigens using artificial circRNAs to trigger adaptive immune responses has exhibited therapeutic and prophylactic effects in coronavirus disease 2019 (COVID-19) and hard-to-treat melanoma malignancies.^27–31^ Compared with the canonical linear mRNA used in vaccines, circRNAs have multiple advantages (Table 1). (1) CircRNAs are more stable and easy to store, whereas mRNA vaccines exhibit extreme instability because it is susceptible to degradation by RNases during transportation, storage, delivery, etc.^22^ Although nucleotide modifications of the mRNA backbone and UTR regions make mRNA more stable, this increases cost and complicates the manufacturing process, and the storage of the resulting vaccine still requires a low-temperature cold chain due to its suboptimal thermostability.^32^ CircRNAs without any modifications exhibit high stability and RNase resistance and can be stored at room temperature or under repeated freeze‒thaw conditions.^27,33–35^ (2) CircRNAs without any modification exhibit fewer side effects. The cytotoxicity and side effects caused by mRNA vaccines are partly due to their high immunogenicity.^36,37^ Compared with modified mRNA, which has somewhat modulated high immunogenicity, circRNA exhibits lower immunogenicity, and lower cytotoxicity in the absence of modification.^38,39^ (3) CircRNAs possess prolonged antigen-yielding capabilities and durable immune responses. The resulting longevity and thus prolonged antigen production contribute to antigen retention in antigen-presenting cells (APCs) and prolong antigen presentation.^40–42^ These factors facilitate the effective triggering of adaptive immune responses and the output of an increased amount of neutralizing antibodies.^27–31^

**Table 1:** Comparison of mRNA vaccine and circRNA vaccine

	mRNA vaccine	circRNA vaccine
Preparation	• Codon optimizations • UTR optimizations • Cap and Poly (A) tail design • Nucleotide modifications • In vitro transcription (IVT) • Purification	• Translation optimizations • Circularization optimizations • Nucleotide modifications • IVT • Circularization • Purification
Storage	• Refrigeration in ultra-cold and RNase-free and sterile conditions	• Non-cryopreservation and preservation under fewer freezing and thawing cycles^28,30^
Biosecurity	• High immunogenicity of unmodified mRNA • Low immunogenicity of modified mRNA	• Low immunogenicity of unmodified circRNA^27,38^ • Lower and even no immunogenicity of modified circRNA^68^
Delivery	• LNP • Polymetric nanoparticles • Cationic nanoemulsion • Exosomes • Solid lipid nanoparticles nanostructured lipid carriers • Naked delivery	• Compatible with mRNA delivery options
Antigens encoding	• Low antigen-encoding endurance due to biodegradation of mRNA	• Prolonged antigen coding tolerances due to the high longevity of circRNA^27,29,30,70^
Application	• Vaccines, encoding adjuvant, encoding antibody, gene editing, and protein replacement	• Compatible with mRNA application. In addition, circRNA can also be applied to sponges and endogenous circRNA mimics

CircRNAs have great potential to become the next generation of RNA-based vaccine platforms. CircRNA vaccines and linear mRNA vaccines have many similarities, especially their ability to express antigens, which depends on both upstream translation initiation and downstream open reading frame (ORF) encoding. Importantly, circRNA can compensate for the multiple disadvantages of mRNA vaccines. However, circRNA vaccines are at an early stage in terms of design, synthesis, purification, delivery, and application. In this review, we summarize the current methodological research on the design of linear RNA precursors, circularization of linear RNA precursors to synthesize circRNAs, purification of synthetic products to enrich circRNAs, delivery of circRNAs to targeted cells, and potential applications of circRNA vaccines. We point out the key issues and challenges facing the use of circRNA as a vaccine, providing insights and perspectives for future investigations.

## The research history of circRNA vaccines

CircRNAs are a class of covalently closed RNA molecules that lack 5’, 3’ ends and polyA tails and were first discovered in plant pathogenic viroids in 1976.^43^ In 1979, circRNA without free flanking ends was also detected in eukaryotic cells using electron microscopy.^44^ In the following decade, circRNA was successively detected in yeast and hepatitis delta virus (HDV), indicating that continuous loop structures of RNA might not be an accidental phenomenon.^45,46^ However, the underlying mechanism of circRNA formation is still unclear. In 1987, circRNA was proposed to form by the ligation of a 5’ splice site to an upstream canonical 3’ splice site, which was formed by splicing of a cryptic 5’ splice site in the 3’ exon.^47^ Compared with canonical splicing during mRNA formation, the above formation pattern led scientists to believe that circRNA might be transcriptional noise caused by the misregulation of RNA splicing.

With the development of sequencing technology, a large number of circRNAs in the mammalian transcriptome were first identified in 2012.^48^ Amazingly, some of the identified circRNAs are even more abundant than their linear counterparts, suggesting that circRNAs may play specific functions in regulating life processes.^49,50^ In 2013, circRNA was reported to function as miRNA sponges, demonstrating that circRNA was equipped with specific regulatory potency.^51,52^ In addition to functioning as noncoding RNAs, circRNAs were reported to guide protein synthesis in 2015.^53^ Furthermore, several circRNA biological functions have been identified, such as interaction with proteins to regulate their activity or translocation or even direct interaction with their host gene to regulate gene expression.^54–57^ With the identification and the functional and mechanistic elucidation of natural circRNAs, they have been increasingly recognized as regulators of cell biology, tumorigenesis, and autophagy, playing important roles in physiological and pathological processes through complex molecular mechanisms.^1–6^

**Fig. 1:**
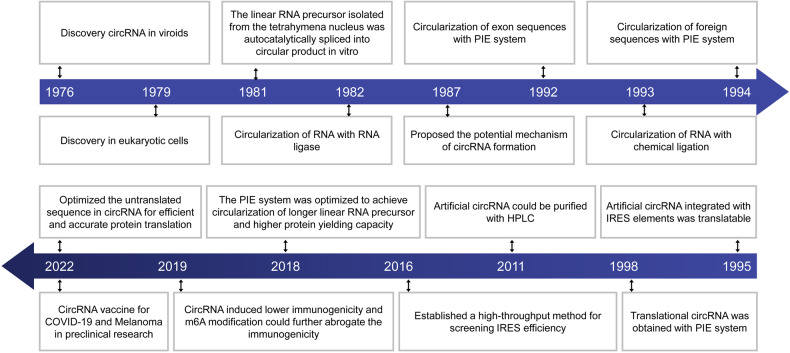
The key discoveries and advances in circRNA vaccines. The development of circRNA vaccine can be divided into three stages. Phase 1, natural circRNA is discovered for the first time,^43^ and the formation mechanism of circRNA is elucidated.^47^ Phase 2 (1981–2022), the development of in vitro synthesis of circRNA. This phase began in 1981. After that, several key advances are developed, including three synthetic method were developed (enzymatic ligation, 1982;^59^ PIE system, 1992;^60^ chemical ligation, 1993^61^), foreign sequences circularization (1994),^62^ introduction of IRES to engineer translational circRNA (1995),^63^ engineering translational circRNA via PIE system (1998),^64^ purification of circRNA by HPLC (2011),^65^ identification of various IRES candidates (2016),^66^ efficient optimization of PIE system (2018),^35,67^ reduction of artificial circRNA immunogenicity (2019) ^38,68^ and achievement of high translation efficiency (2022).^69^ Phase 3 (2022~), engineering circRNA vaccines are used for the first time in the treatment and prevention of COVID-19 and melanoma.^27–31^ Future development will continue to advance

The discovery of circRNA in organisms prompted the exploration of the properties and functions of circRNA through in vitro synthesis. In vitro circularization of a linear RNA precursor was disclosed in 1981, when a linear RNA molecule from an isolated transcript in the nucleus of a tetrahymena cell was autocatalytically spliced into a circular product.^58^ This was the cornerstone of later group I intron-mediated in vitro synthesis of circRNA. In 1982, researchers performed the enzymatic circular ligation of linear RNA by RNA ligase.^59^ In 1992, researchers constructed a permuted intron-exon (PIE) system by utilizing tetrahymena group I introns, which contained end-to-end fused exons to interrupt half introns. This system can ligate exon sequences through an autocatalytic splicing reaction to form circular products.^60^ In 1993, circular single-stranded nucleic acids were ligated using chemical coupling agents (chemical ligation).^61^ In that context, artificial circRNA was engineered in 1994, when foreign sequences could be placed in the exon and made circular in vitro using the PIE system, which was constructed by the group I intron of the thymidylate synthase (td) genes of T4 bacteriophage.^62^ This proved the possibility of engineering circRNA in vitro. Furthermore, researchers demonstrated that the internal ribosome entry site (IRES) and continuous ORF could drive the translation of artificial circRNA in 1995.^63^ Although it was possible to ligate linear RNA to form translatable circRNA using T4 DNA ligase, it required terminal modification and a complex process that did not suit the synthesis of circRNA.^63^ Most importantly, in 1998, the PIE system successfully ligated a linear RNA precursor containing a simple green fluorescent protein (GFP) ORF, and this artificial circRNA correctly expressed GFP in E. coli.^64^ This strategy achieves the in vitro synthesis of an artificial translatable circRNA, although the translational efficiency of circRNAs is poor when compared with a similar linear RNA species. In 2011, it was found that in vitro synthesis of RNA produces large amounts of contaminants, and the use of high-pressure liquid chromatography (HPLC) technology enables the purification of the products, which reduces exogenous nucleic acid-induced innate immunogenicity and enables in vivo experiments.^65^ In 2016, systematic high-throughput methods for discovering and characterizing IRESs in human and viral genomes were established, providing the basis for subsequent IRES selection and translation improvement.^66^ Remarkably, starting in 2018, emerging studies optimized the PIE system to achieve feasible circularization of longer in vitro transcription (IVT) linear RNA precursors and achieved higher protein-yielding capacity.^35,67^ In 2019, further study demonstrated that circRNA exhibits low immunogenicity and m6A modification in exogenous circRNA can almost abrogate its immunogenicity.^38,68^ In 2022, researchers re-optimize the untranslated sequence in circRNA for efficient and accurate protein translation.^69^ These developments have led to breakthroughs in the artificial in vitro synthesis of circRNA and make engineering circRNA possible as a biomedicine strategy (Fig. 1).

Given that artificial circRNA can correctly express proteins in mammalian cells and exhibits low immunogenicity, purified artificial circRNA may be suitable for in vivo applications such as vaccines or therapeutic medicines. In 2022, engineered circRNAs were utilized to express relevant antigens to trigger adaptive immune responses and disease therapeutic effects, for example, encoding the binding domain (RBD) of the SARS-CoV-2 spike protein to inhibit SARS-CoV-2 virus,^27,28,30,31^ encoding chicken ovalbumin (OVA) to treat melanoma malignancy,^29^ and encoding cytokines such as IL-15, IL-12, GM-CSF, and IFN-a 2b to facilitate anti-PD-1 antibody-mediated tumor suppression.^70^ It is worth mentioning that in addition to being translated into proteins for disease prevention and treatment, artificial circRNA can also play an antitumor role through noncoding functions. For example, in 2018, an in vitro-synthesized circRNA containing multiple miR-21 bulged binding sites effectively inhibited miR-21 activity and gastric cancer cell proliferation compared with a miR-21 inhibitor.^71^ After that, various artificial circRNAs emerged to act as endogenous miRNA sponges.^72–74^ However, these circRNA-based therapeutic platforms do not maintain a long-lasting therapeutic effect and do not have the prophylactic effect that a vaccine should have. Thus, the emergence and development of circRNA vaccines is still in the preliminary stage.

## Design of linear RNA precursors

**Fig. 2:**
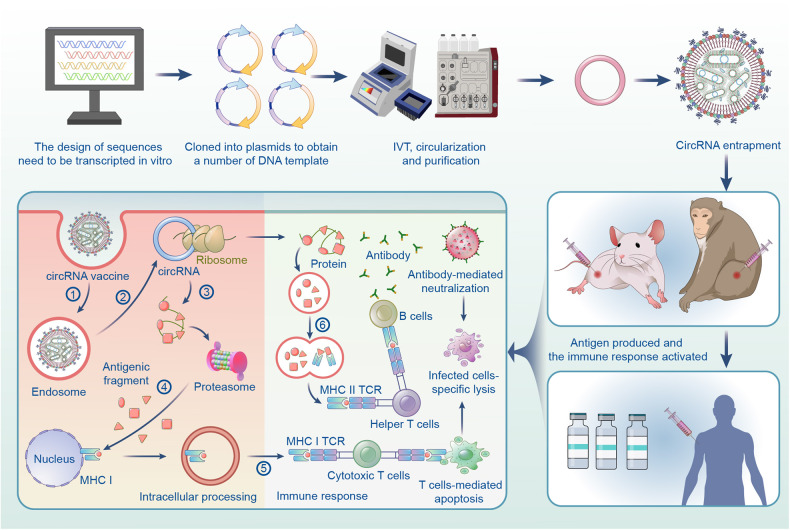
Establishment of circRNA vaccine production pipeline and circRNA vaccines to elicit immunity by utilizing disease-specific targeted antigen strategies. Briefly, The encoding sequence of peptide/protein is designed according to the intended antigen and cloned into a plasmid DNA construct. Plasmid DNA is transcripted into linear RNA precursor (pre-circRNA) by in vitro transcription (IVT) technology. Then the pre-circRNA is cyclized as circRNA in vitro. And circRNA is further purified by high-performance liquid chromatography (HPLC) to remove contaminants and reactants. Subsequently, purified circRNA is entrapped in various vehicles. Prior to clinical trials, pharmacodynamic and biosafety evaluation should be conducted. Finally, the scale-up manufacturing of circRNA vaccine is followed by the clinical trial. In the induction of antigen-specific immune responses. The circRNA vaccines mainly follow three aspects in intracellular processes, including endosome escape, antigen encoding, and immune initiation. Briefly, In antigen-presenting cells (APCs), (1) LNPs containing circRNA vaccines form endosomes in the cytoplasm. Then, (2) endosomes release the circRNA vaccine (endosome escape). Subsequently, (3) encoding sequences in circRNA were translated into antigenic proteins/peptides by ribosomes. (4) endogenous antigens are degraded into polypeptides by the proteasome and are presented by MHC I and (5) activate cytotoxic T cells (CD8^+^ T cells). In addition to cellular immunity, circRNA-induced humoral immunity is also important for disease prevention. (6) endogenous antigens in APCs can be secreted and be presented to the helper T cells by MHC class II proteins. Helper T cells (CD4^+^ T cells) stimulate B cells to produce neutralizing antibodies

**Fig. 3:**
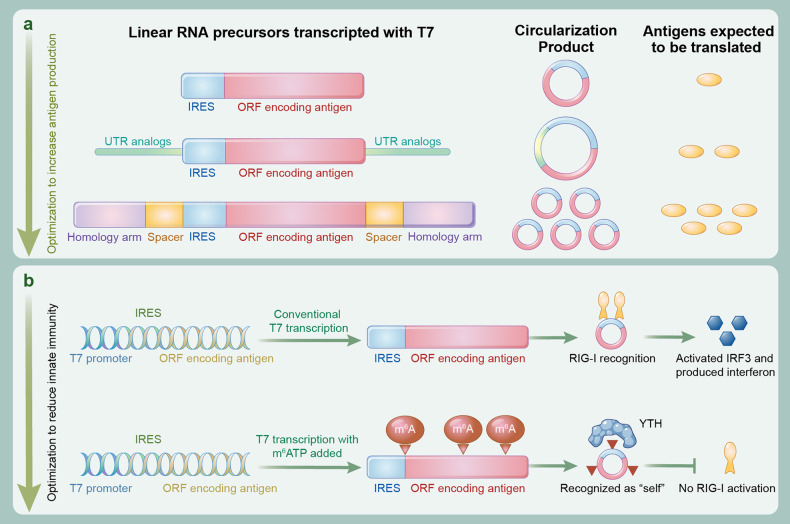
Design of linear RNA precursor (pre-circRNA) for efficient circularization, translation initiation and avoidance of immunogenicity. **a** The untranslated region (UTR) containing the RNA-binding proteins (RBPs) motif can be added upstream and downstream of the IRES-ORF cassettes of linear RNA precursor to promote translation efficiency, and homologous arms and unstructured spacer sequences can be added to promote circularization and to further improve translation efficiency. **b** During in vitro transcription (IVT), nucleotide modification can be achieved by the addition of N6-methyl-ATP (m6ATP) to the reaction buffer, which allows the host to recognize the circRNA as its own, significantly reducing or even abrogating the immunogenicity of circRNAs

The preparation of circRNA vaccine mainly includes circRNA design, synthesis, purification, circRNA entrapment, pharmacodynamics, safety evaluation, manufacturing, and clinical trials (Fig. 2). Linear RNA precursors (pre-circRNA) to circularization need to be designed to contain diverse elements to comprise entire vaccine features and functions. CircRNA vaccines rely on the translation system of host organisms to produce antigens, which in turn stimulate the immune response. Therefore, the ORF encoding the antigen and the elements mediating its translation are necessary for the design of linear precursors. Taking antigen reactiveness into consideration, the above basal elements should also be optimized to improve translational efficiency. More importantly, the elements facilitating circularization and reducing immunogenicity are also crucial. Multiple design strategies should be synchronized to confer the superiority and feasibility of circRNA as a therapeutic platform (Fig. 3a).

IRES-ORF cassettes are core elements in linear RNA precursors. The canonical translation model in eukaryotic cells is the 5’ -cap-dependent type. For this type, translation initiation involves binding of the 7-methylguanylate (m^7^G) cap structure at the 5’ -UTR to eukaryotic initiation factor 4E (eIF4E), which, in synergy with eIF4A and eIF4G, scaffolds other initiation factors and ribosomes.^75^ In contrast, circRNAs are covalently linked head to tail and lack a 5’ terminus, and their translation must rely on cap-independent mechanisms.^63^ Cap-independent translation is an alternative means of translation initiation in eukaryotes that depends on the presence of particular elements that induce internal translation initiation. IRESs are special sequences located upstream of the start coding codons, which enable the engagement of 40 S ribosomal subunits to induce downstream read and encode in a 5’ cap-independent manner.^53,76^ In addition to IRES, ORF elements encoding customized proteins/peptides downstream of IRES are crucial for storing coding information. Encoding of the protein/peptide can be initiated when 40 S ribosomal subunits engage in the start coding codons of ORF and finish at the termination codons of ORF.^77–80^ This process will be repeated as long as circRNA is not been degraded. Notably, IRES-mediated translation should occur readily through upstream ORFs without any scar sequences between the IRES and ORF, otherwise resulting in reduced translation efficiency.^69^ Therefore, designing sequencing elements to mimic the highly structured IRES and proper ORF is the primary method for engineering circRNA vaccines. Furthermore, canonical IRES types that have been used for circRNA translation are mainly derived from poliovirus 1 (PV1), human rhinovirus A1 (HRV-A1), encephalomyocarditis virus (EMCV), hepatitis C virus (HCV), cricket paralysis virus (CrPV) and coxsackievirus B3 (CVB3). It is worth noting that although the IRES from CVB3 has been demonstrated to be superior in initiating circRNA translation, the comparison with the human IRES mentioned above has not been reported.^35^ In addition, IRES efficacy varies depending on cell type and species, and research and application need to be re-examined.^35^

A customized IRES or ORF may further expand the translation efficiency. Although CVB3 IRES is considered to have a higher translation-initiating ability, its excessive length, coupled with that of the ORF, significantly increases secondary structure formation and circularization pressure. In addition, long circRNAs are more prone to nicking.^35^ How to shorten the circRNA is an important question for future investigation. For this reason, screening of shorter IRES analogs, such as Kozak sequences and AU-rich sequences, may eliminate these restrictions.^78^ More broadly, like IRES, the ORF is typically above 1000 nt, and together with the requirement to express combined or fused antigens, this will significantly increase the linear precursor length. There has been increasing attention given to the idea that short ORF (sORF) or customized ORFs to express neoantigens with lengths ranging from 7 to 11 amino acids may confer the expected immune effects.^81^ The identification of a sORF or a customization of an ORF to replace a long ORF, which, in synergy with sIRES, may enable the use of a shorter and more flexible circRNA. There are many strategies to identify sORFs. Examples include cross-species comparisons to identify conserved sequences, the examination of codon content or features to differentiate potential coding sORFs, and translation approaches to identify coding sORFs.^82^ Moreover, the best tool for exploring sORFs is ribosome profiling (library construction), which is broadly applied in identifying ORFs in circRNAs.^83^

Specific motifs upstream and downstream of IRES-ORF cassettes increase translation efficiency. The 5’ and 3’ UTRs in mRNAs can recruit RNA-binding proteins (RBPs) that enable translational initiation.^84–88^ Although circRNAs do not have any UTR regions, regions located upstream and downstream of the IRES-ORF cassettes can be designed as UTR analogs. Therefore, these regions can be designed with specific motifs to imitate the binding sites of RBPs. For example, poly(A)-binding protein (PABP) motifs are introduced to trigger the binding of eIFs, poly(C)-binding protein (PCBP) motifs are introduced to recruit ribosomal proteins and trans-activating factors, and m6A motifs (RRACH, R = G or A; H = A, C or U) are introduced to induce the recruitment of YTHDF3 and translation initiation factors eIF4G2 and eIF3A.^89–91^ A synthetic IRES containing an eIF4G-recruiting aptamer also drives stronger circRNA translation.^69^ Reportedly, translation of circRNAs can be improved by the addition of 50-nt spacers which separates the IRES and interested gene from the splicing scar and changes the topology of the circRNA vector.^69^ Moreover, the addition of a 50-nt region upstream and downstream of IRES-ORF cassettes for costuming RBP motifs, such as the PABP motif upstream and HBA1 downstream, can further increase translation efficiency to different levels.^69^ Unlike mRNA, inserting the PABP motif or poly(T) downstream of the IRES-ORF cassette region reduces protein production.^53^ However, the incorporation of nucleotides has been shown to alter the secondary structure, thereby resulting in molecular instability or eliminating the binding ability of other proteins.^92^ To overcome this limitation, the rational design of minimalistic protein-binding motifs may be promising for improving circRNA translation. Hence, the regions upstream and downstream of IRES-ORF cassettes may be the ideal operational floor in translation regulation, and their diminished length and incorporation of specific motifs may trigger positive translation efficiency.

Nucleotide m6A modifications may reduce circRNA immunogenicity. The innate immune system relies on pattern recognition receptors (PRRs), which mainly include Toll-like receptors (TLRs) and retinoic acid-inducible gene I (RIG-I)-like receptors (RLRs). Among them, RIG-I is a critical cytosolic RNA sensor in the detection of single-stranded RNA (ssRNA).^93,94^ The activation of RIG-I provides intact IFN pathways and concomitant hyperproduction of cytokines, leading to cytokine storm or cytokine release syndrome and severe inflammation.^95–98^ It was recently reported that the immunogenicity of unmodified circRNA after purification efficiently is negligible.^38^ However, many studies have proven that in vitro-synthesized circRNAs themselves induce innate immunity responses, particularly the circRNA with exogenous sequences.^29,30,35,99^ This means that circRNAs must be designed as endogenous nucleic acids to escape surveillance by the innate immune system. Evidence indicates that m6A is the most abundant modification on natural mRNAs and is present on 0.4–0.6% of all adenosines in mammalian polyA-tailed transcripts.^100^ Consistently, m6A-modified natural circRNAs are abundant according to m6A-methylated RNA immunoprecipitation sequencing (MeRIPseq) and m6A-circRNA microarray data.^100,101^ As expected, 1% m6A modifications in artificial circRNA sufficed to reduce the induction of innate immunogenicity, whereas 100% m6A modifications in artificial circRNA completely abrogated the induction of innate immunogenicity. Further mechanistic studies have shown that m6A modifications in circRNA recruit its cytoplasmic reader YTHDF2 to install endogenous compounds^89^ (Fig. 3b). These results suggest that exogenous artificial circRNA requires binding to cytoplasmic RBPs to disguise its foreign role. Considering the large RBP population in vivo, designing multiple RBP motifs or nucleotide modifications in linear precursors can incorporate artificial circRNA into endogenous nucleic acid populations.

The homology arms and permissive spacers promote subsequent circularization processes. As the most commonly used circularization method, the splicing efficiency of the group I intron-assembled PIE system is inherently low (this will be discussed in the next section). For several reasons, the length between the 5’ and 3’ splice sites of the native group I intron averages 300–500 nt, and yet in vitro preparation of linear RNA precursor containing complex elements significantly extends the length.^102^ Long intervening regions between splice sites interfere with the formation of a stable complex, which may reduce the ability of the splice sites to interact with each other and thus reduce splicing efficiency. Considering this, in linear precursors of 5 kb in length, the addition of external and internal complementary homologous arms at the 5’ and 3’ ends (excised intron fragments) and 5’ and 3’ splice sites (resided exon fragments), respectively, can significantly improve the circularization efficiency.^35^ Furthermore, single-stranded RNA precursors can form complex secondary structures, especially highly structured IRES and ORF elements, which decrease folding of the splicing ribozyme and interfere with normal splicing reactions. Hence, the insertion of spacer sequences that are unstructured and nonhomologous at the splice site to distance the splice site from the proximal IRES-ORF cassette can significantly promote the folding of the splicing ribozyme and increase the circularization efficiency.^35^ Additionally, the separation of the splice site and IRES-ORF cassettes allows them to fold and function independently, improving the efficiency and accuracy of circRNA encoding. Likewise, the other circularization methods are applicable to these optimization measures. The introduction of spacers with free secondary structures at the ends of the two flanks may also promote the circularization efficiency and the functions of circRNA.

## Synthesis of circRNA vaccines

**Fig. 4:**
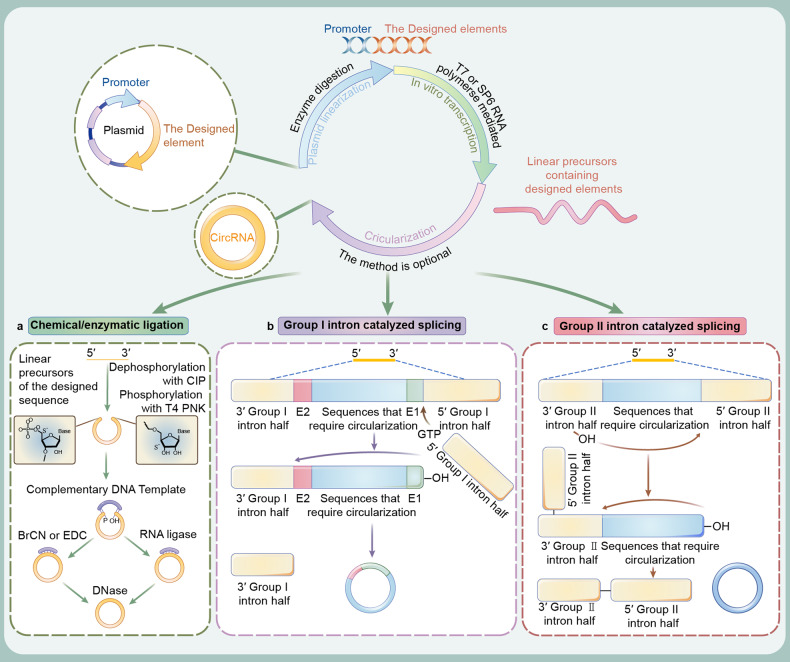
Schematic illustration of in vitro transcription (IVT) technology and circularization of linear RNA precursor (circRNA synthesis). The process of IVT include plasmid construction, plasmid linearization and IVT (production of linear precursors). **a** Chemical ligation methods by the conjugation of 5’-end phosphate with 3’-end hydroxyl catalyzed by BrCN or EDC treatment. Enzymatic ligation method, catalyzed by RNA ligases, use complementary template to bridge specific ligations in two flanks. And then the DNase strips the template. **b** Group I intron-based permuted intron‒exon (PIE) system. Permutation of a native group I intron and insertion of a custom sequence into the exonic region (E1 and E2). And then this PIE system spontaneously ligates in the presence of free guanosine, making it circular and freeing the two half-intron fragments. **c** Group II intron-based PIE system. The custom sequence is flanked by the 5’- and 3’-self-cleaving native group II intron. And then, this system automatically ligates and excise lariat intermediate

Linear RNA precursors to circularization are commonly synthesized using the IVT method (Fig. 4). This method utilizes a bacteriophage RNA polymerase, such as T7 or SP6 RNA polymerase, to amplify the target sequence in the DNA temple to produce large numbers of linear precursors. It is undoubtedly simpler, quicker and cleaner than large-scale protein production and purification. For the synthesis of nucleotide-modified linear RNA precursors, such as m6A, amplification buffer can be added with N6-methyl-ATP (m6ATP).^103^ After IVT synthesis, the method of linear precursor circularization is optional. In vivo, natural circRNAs are spliced from lariat-driven circularization, exon skipping-driven circularization, intron-pairing-driven circularization, directed back splicing, tail cutting-driven intron circularization, and RBP- or trans-factor-driven circularization.^104^ The natural ligation site is generated by connecting the 3’-OH splice acceptor and 5’-phosphate splice donor with a covalently 3’, 5’-phosphodiester bond.^105^ For circularization of linear RNA precursors in vitro, which splice their two flanks, conditions such as approximation of two flanks, catalysts of substances, or natural splicing systems should be created. Based on current studies, chemical, enzymatic, and ribozyme ligation are the main approaches to circularization of linear RNA precursors (Fig. 4 and Table 2).

**Table 2:** The advantage and disadvantage of the three circularization methods

	Advantage	Disadvantage
Chemical synthesis strategies	• No exogenous sequence introduction	• Low circularization efficiency for long linear precursor • Complicated design and synthesis procedures • Complementary template requirement • Low-throughput synthesis • Unknown immunogenicity
Enzymatic synthesis strategies	• No exogenous sequence introduction • Lowest immunogenicity	• Low circularization efficiency for long linear precursor • Complicated synthesis procedure • Complementary template requirement • Low-throughput synthesis
PIE system	• Simple synthesis procedure • Suitable for circularization of long linear precursor • Applicable to high-throughput synthesis • Low immunogenicity	• Exogenous sequence introduction (group I intron PIE system) • Contaminants present after synthesis

In chemical synthesis strategies, the 5’-terminal phosphate and 3’-terminus hydroxyl group provide the functionality requirement for ligation, and some condensation reagents, such as cyanogen bromide (BrCN), 1-ethyl-3-(3-dimethylaminopropyl) carbodiimide (EDC), and azide-alkyne cycloadditions (ACC), are needed to facilitate chemical ligation^106,107^ (Fig. 4a). Chemical amino modification was conducted at the 3’-terminus of the linear precursor to mediate circularization. However, the above chemical strategies generate 2’, 5’phosphate bonds and phosphoramidite (P-N) bonds, neither of which are natural 3’, 5’phosphodiester bonds. Even worse, the chemical strategies both suffer from low circularization capacity, probably because it is harder to bring the two termini together for ligation when the RNA is longer.^106^ Concerning this, further conjugation of a complementary oligonucleotide template for two flanking sequences can conglutinate two ends of the linear RNA precursor, improving the circularization efficiency.^61^ The template can be digested by DNase after ligation. Although circRNAs ligated by such a method can serve as translation templates, unnatural linkages may have potential biosafety problems.^108,109^ Recently, the applications of click chemistry, which has fast reaction kinetics, quantitative yields, minimal byproducts, and high chemospecificity, biocompatibility and regioselectivity, may overcome the inherent defects of chemical synthesis strategies.^110,111^ Further studies, especially in vivo experiments, are also required to ascertain the above questions.

Enzymatic synthesis strategies are commonly achieved by RNA ligases (Fig. 4a). Such ligases include T4 RNA ligase I and T4 RNA ligase II, which are created from bacteriophage T4-infected *Escherichia coli*.^112^ T4 RNA ligases catalyze the formation of a covalent 3’, 5’-phosphodiester bond between 5’-phosphate and 3’-hydroxyl end groups in an ATP-dependent manner.^113^ Among them, RNA ligase I can achieve high-efficiency single-stranded RNA linkage, but it is only applicable to linear RNA precursors smaller than 500 nt, and the end of linear RNA precursors should be free of secondary structure.^71,114,115^ Moreover, intermolecular linkages can form oligomers due to the lower reaction specificity of RNA ligase I.^116^ Herein, T4 RNA ligase I is not suitable for circularization of linear precursors. Notably, RNA ligase II has higher activity, sensitivity and ligation efficiency because of its ability to repair a wide range of RNA damage in vivo.^117^ During the closure of the nick, RNA ligase II binds the 3’-OH side of the linear precursor. Then, adenylated RNA ligase II transfers the adenosine group to the 5’-phosphate. Finally, adenosine is attacked by 3’-OH to complete the formation of the phosphodiester bond. However, the sensitive character can cause intermolecular ligation, producing significant amounts of polymeric byproducts.^118^ Hence, similar to chemical synthesis strategies, the conjugation of complementary hybridized oligonucleotide templates is conducive to individually bridging two ends of a single-stranded precursor together.^119–122^ Fortunately, T4 RNA ligases producing circRNA without extraneous fragments exhibit minimized innate immunogenicity.^123^ They have more potential as a circularization approach for linear precursors. Further optimizations are suggested, such as the introduction of unstructured elements without complex secondary structures at the ends of the two flanks or the introduction of homologous spacers that can be modified.

Ribozyme ligation is the most common circularization approach and is achieved by PIE system-mediated consecutive ester exchange reactions. Group I introns, which consist of fused partial exons flanked by half-intron sequences, are autocatalytic ribozymes that ligate long linear RNA precursors containing customized target sequences through self-catalyzed splicing reactions.^59,124^ Group I intron catalysis of cyclic ribonucleotide ligation is a commonly used PIE cyclization strategy that assembles the pre-tRNA_leu_ and thymidylate synthase (td) genes of the cyanobacteria Anabaena and T4 bacteriophage, respectively, and only requires the addition of GTP and Mg^2^^+^ as coreactors.^67^ Group I intron self-splicing reactions occur through two consecutive ester exchange reactions. During splicing, exogenous GTP binds to the G-OH conjugation site at the 5’ splice site. G-OH engages in a transesterification reaction, forming exon 1 (E1) with a free 3’-OH group and excised 5’ intron with exogenous G. Subsequently, the freed 3’-OH group engages in a second transesterification at the 3’ splice site, resulting in excision of the 3’ intron as well as ligation of exon 2 (E2) and E1^102^ (Fig. 4b). Moreover, engineered circRNA regulators (ECRRs) may bind to linear precursors and form homodimers to promote ligation.^125^ Some cooperating proteins, such as RBPs, have been identified as back-splicing regulators. For example, muscleblind (MBL) binds to the sites inside the flanking intron splice sites of linear RNA and promotes back splicing, and the PUF domain of ECRRs was designed to specifically recognize the same 8 nt target sequences both upstream and downstream of the linear RNA precursor.^7^ Therefore, the design of RBP motifs in linear precursors and the use of RBPs during in vitro circularization processes can greatly facilitate circRNA ligation as efficiently as the addition of homology arms.

However, a limitation of the group I intron self-splicing reaction is that RNA fragments (E1 and E2, called scar sequence) larger than 80 nt reserves in the end-joining sites of circRNA. This limitation may contaminate the target sequences, even causing them to differ from their original linear RNA precursor.^123^ Accordingly, new strategies should be designed. Some strategies identify the ligation site.^53,126^ This suggests that ORF or multiple IRES candidates can be modified or mutated to fit the ligation site sequence, thereby concealing E1 and E2 into functional sequences. Compared with the group I intron PIE system, group II introns can be used for artificial circRNA circularization without residual exogenous sequences, thus producing a more accurate sequence mimic. Group II introns are retrotransposable elements derived from bacteria or organelles of fungi, algae and plants.^127^ These ribozymes initiate autocatalytic splicing reactions. Initially, 2’-OH in the 3’ intron nucleophilically attacks the 5’-splice site, yielding a lariat intermediate with a 2’, 5’ branch structure. Hereafter, the 2’-OH group in the 3’ end attacks the 3’ splice site of the lariat intermediate, resulting in excision of the lariat intron and ligation of flanking exons^128–130^ (Fig. 4c). Self-splicing group II introns enable efficient circularization of scarless circRNAs, and this artificial circRNA can be customized to contain different ORFs to direct translation.^27^ The construction of a PIE method without exogenous sequences is still currently a challenge to be addressed.

Other circularization methods utilizing 3’ intron remodeling may provide favorable yields in inducing circRNA formation. For example, a hairpin designed at the 3’ intron can be targeted and removed by the CRISPR endoribonuclease Csy4, exposing the splice signals and inducing back splicing.^131,132^ The build of “Twister” aptamers in the 3’ intron may achieve wide linear RNA circularization. Mechanistically, tRNA endonuclease recognizes “Twister” aptamers to generate a 2’, 3’-cyclic phosphate group at the 5’ terminus.^133^ Subsequently, the ubiquitous endogenous RNA ligase RtcB connects the 3’ terminus and 5’-OH.^134,135^ It is worth mentioning that these methods involve sophisticated steps and are not suitable for the large-scale industrial preparation of vaccines.

After circRNA synthesis, structure identification should be performed. The ligation site, which is the head-to-tail junction donated by the two flanks of the linear RNA precursor, is present in the circRNA but not in the linear RNA precursor. Thus, after reverse transcription and cDNA synthesis, a pair of primers spanning the region containing the ligation site can be used to identify the presence of circRNA by using PCR.^35^ Furthermore, the molecular size of the above PCR products can be verified by gel electrophoresis. Sequencing of the product can further determine the successful synthesis of circRNA.^32^

## Purification of circRNA vaccines

The purity of circRNA is important because both the low immunogenicity and protein expression depend on the absolute homogeneity of circRNA components.^35^ However, the PIE method, which has advantages in synthesizing larger linear RNA and simpler reactions, can generate extra polymerization side products, including free-style excised intron fragments, remnants of linear precursors, triphosphate-RNA, polyadenylated RNA and nicked circRNA, which is an open-loop isoform.^65^ T4 RNA ligase also leaves large numbers of split templates that are difficult to remove. These linear RNAs, as ligation reaction contaminants, are considered strong immunogens that can be detected by PRRs and lead to innate immune responses. Therefore, thorough purification of circRNA preparations and detection of circRNA purity are essential.

**Fig. 5:**
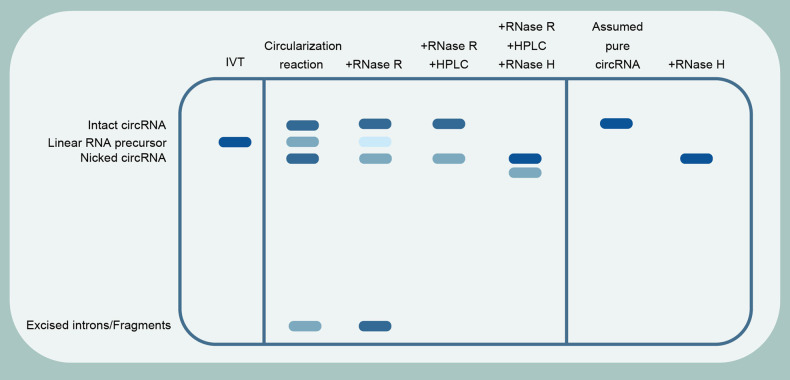
The hypothesis of purification results for group I intron permuted intron‒exon (PIE) system by E-Gel EX Agarose Gels. Splicing reaction by group I intron PIE system can produce several impurities. RNase R can digest a portion of linear RNA and nicked circRNA. Subsequently, HPLC can eliminate most of the fragments except for impurities of the same size. In addition, RNase H is the best option to detect the purity of circRNA products. The pure circRNA products exhibit only one bond and impure circRNA products exhibit two bonds

Owing to the size discrepancy of products synthesized in vitro, electrophoresis is a routine approach to verify the compositions (Fig. 5). Denaturing agarose gel electrophoresis (DAGE) and capillary electrophoresis (CE) are economical methods commonly used to identify RNA species after IVT, as RNA mobility is roughly inversely proportional to its molecular size.^136,137^ DAGE and CE both reflect the amount and percentage of circRNA.^70^ Moreover, polyacrylamide gel electrophoresis (PAGE), which is formed by the polymerization of acrylamide in the presence of a cross-linking reagent, allows separation of nucleic acids according to the size of the molecule. PAGE with gel purification is commonly used to remove undesired products to obtain desired RNA samples since RNAs exhibit different migration patterns from their counterparts.^138^ DAGE and PAGE allow the simple identification of circular splicing products from linear precursor molecules, excised introns, and nicked circRNA, which is a nonspecifically nicking contaminant generated by the application of Mg2^+^ and heat. Of note, although the three synthesized products (linear precursor molecules, nicked circRNA, and intact circRNA) are equal in molecular weight, their different secondary structure determines their migration rate during electrophoresis. Hence the bands representing linear precursor molecules, nicked circRNA and intact circRNA are easily distinguished in electrophoresis. Evidence indicated that intact circRNA migrated more slowly than equal-weight nicked circRNA in EX-based E-gels, a neoteric electrophoresis system with SYBR-GOLD II as a detection dye and GLBII as a running buffer.^38,139^ This novel electrophoresis system can partially enlarge the distance of the bands representing linear precursor molecules, nicked circRNA, and intact circRNA, but is expensive and may show variability between lots. Fortunately, to verify the intact circRNA ratio, intact circRNA can be specifically nicked by using oligonucleotide-guided RNase H degradation. After nicking, intact circRNA molecules will linearize and generate a single band, whereas nicked circRNA and linear RNA precursors will yield two bands on gels.^35,134^ Overall, the use of electrophoresis methods allows for simple and effective discrimination of circRNA from linear RNA precursors, nicked circRNA, and splicing intermediates such as excised introns. However, regarding vaccine applications, electrophoresis methods are not appropriate for large-scale purification, and electrophoresis generates heat in the gel and running buffer that may affect RNA stability. Therefore, electrophoresis methods are suitable only for application to various quality tests, such as quantity, purity and identity tests for in vitro-synthesized circRNA products.

Treatment with RNase R to eliminate the linear RNA contaminants. After circRNA synthesis, circRNA is a unique ring-shaped product. Therefore, the degradation of all linear RNAs using the distinctive properties of circRNA is the principal method for enriching circRNA. CircRNAs are more stable than linear RNA and resist exonuclease digestion due to their closed-loop structure; thus, in laboratory experiments, linear RNA is degraded by treating RNA samples with exonucleases such as RNase R to identify circRNA in transcripts.^140,141^ However, RNase R with 3’ to 5’ exonuclease activity requires RNAs with at least a 7 nt single-stranded 3’ overhang, which suggests that linear RNAs with extensive secondary structure in the 3’ end region are resistant to RNase R.^142^ Moreover, some regions, such as G-rich forming G-quadruplexes with intricate secondary structures, can abruptly stall the migration of RNase R, producing truncated linear RNAs.^143^ Hence, eliminating secondary structures in linear RNAs can enhance their sensitivity to RNase R. Because the formation of G-quadruplex structures is K^+^ dependent, the presence of K^+^ in the buffer should be avoided or replaced by other ions in the reactive buffer.^144^ In general, the major obstacle to RNase R digestion is the low sensitivity of linear RNA precursors possessing complex secondary structures, which is one of the reasons for incomplete subsequent purification. Although many methods for the disassembly of RNA secondary structures are available, such as high-temperature heating and incubation with chemical reagents, their disassembly efficiency and compatibility in subsequent enrichment reactions are unknown. Given the lack of single-stranded overhangs at the 3’ end linear RNAs are more resistant to RNase R degradation, linear RNA precursors can incorporate elements without secondary structure as a way to increase sensitivity to RNase R. For example, combining the introduction of poly(A) tails with the treatment of RNase R enables a general improvement in the degradation efficiency of linear RNA.^143^ Furthermore, circRNAs are not completely resistant to RNase R. Evidence demonstrates that prolonged exposure to RNase R can also degrade circRNAs due to the formation of spontaneous nicks in circRNAs over time and due to the inherently unstable nature of RNAs.^145^ High concentrations of RNase R also cause degradation of circRNA, but low concentrations of RNase R may not adequately degrade linear RNAs.^139,140,146^ The incubation time and reaction conditions need to be optimized. These results illustrate the multiple disadvantages of using RNase R to enrich circRNA. Better methods for the enrichment of circRNA are needed.

High-performance liquid chromatography (HPLC) is a chromatographic technique for rapidly and efficiently fractionating an RNA mixture. Based on differences in the molecular sizes and polarities of individual species, HPLC can separate multicomponent samples with strong sensitivity, high precision, broad range, and massive sample throughput.^147^ Moreover, as a chromatographic method, size-exclusion chromatography (SEC) allows the separation of molecules according to their size. SEC is a slow method requiring a long column. Its resolution is very limited due to the band-spreading effect, especially during long operating times. SEC combined with HPLC (SE-HPLC) has been employed for rapid and selective characterization of influenza virus vaccine constituents.^148,149^ An advantage is that SEC-HPLC easily removes smaller free-style intron fragments. However, the separation ability may be reduced in the separation of compositions with little difference in molecular size. By combining SEC-HPLC with RNase R digestion, the property gaps in circRNA, nicked circRNA, linear RNA precursors and other products are enlarged, which can further improve the separation efficiency of HPLC, with 90% circRNA.^35,139^ This is the most efficient method of purification at present.

How to obtain pure circRNA remains an unsolved problem. Although splicing reactions followed by RNase R digestion and additional HPLC purification can diminish some immunogenic RNA species, they are not sufficient to prevent the innate immune response and release of cytokines.^38^ Hence, the HPLC purification of circRNA presents unique difficulties. On the one hand, it is difficult to distinguish compounds with similar structures, sizes and polarities by HPLC. Nicked circRNAs, linear RNA precursors and intact circRNAs are equal in molecular weight, and their respective peaks partly overlap. Unfortunately, due to its high structure, RNase R digestion cannot completely digest nicked circRNA.^35,38^ Linear RNA precursors with secondary structures may increase resistance to RNase R.^142,143^ As demonstrated by gel electrophoresis, the nicked circRNA and linear RNA precursor resided after RNase R digestion and HPLC purification.^35,38^ Interestingly, absorbance traces show that intact circRNA is smaller than nicked circRNA of equivalent molecular size, and an apparently higher-molecular-weight nicked peak emerges after circRNA digestion by RNase H.^139^ Perhaps this distinction can be used to separate intact circRNA and nicked circRNA. This result indicates that ensuring the robustness of peak separation is one of the most important parameters of HPLC purification. On the other hand, alkaline phosphatase treatment after HPLC purification and before RNase R digestion dramatically reduces the innate immune response.^38^ This means that some side effects of pure methods, such as heat exposure or storage options, may lead to product instability, resulting in massive residual triphosphate and divalent cations, which is another factor that provokes robust cellular immune responses. And some residual linear RNAs that expose triphosphorylated tails after HPLC purification are stronger immunogenic. Overall, the current RNase R digestion and additional HPLC purification for circRNA purification are still insufficient. The purification methodology should be further improved.

## Delivery of circRNA vaccines

In addition to purification to ensure that circRNA vaccine components are homogeneous and do not elicit an immune response in vivo, a suitable delivery system can also enable circRNAs to evade surveillance by the autoimmune system and can help deliver circRNA vaccines to their target sites for expression. However, the development of RNA-based vaccine therapeutics is hindered by difficulties in their cellular delivery. For example, nucleic acids can interact with serum proteins, be taken up by phagocytes, and be degraded by endogenous nucleases.^150^ More importantly, the negative charges and hydrophilicity prevent nucleic acids from passing through the anionic lipid bilayer of cell membranes.^151,152^ Thus, the delivery system plays a key role in effectively protecting exogenous circRNA vaccines and transporting them to cells. Several innovative delivery systems have been developed. This section will introduce the delivery strategies of circRNA vaccines based on relevant studies, limited by the early stages of the circRNA vaccine field, and will also discuss potential strategies.

### Lipid-based delivery

Liposomes are pioneering nanomedicine delivery platforms because they are very versatile nanocarriers that can transport hydrophobic or hydrophilic molecules.^153^ As the field develops, it has been generally accepted that smaller liposomes have higher chances of escaping phagocyte uptake and entering targeted cells.^154^ Stabilized lipid-based nanoparticles (LNPs) with sizes ≤100 nm are a new generation of liposomes, including solid lipid nanoparticles, nanostructured lipid carriers, and cationic lipid amphiphiles, that exhibit improved internal physical stabilities and the ability to control the location and timing of delivery in vivo.^155^ Therefore, LNPs with favorable biocompatibility and in vivo delivery capacity as promising vehicles have emerged across the biomedical industry to deliver therapeutic nucleic acid preparations.

Cationic ionizable LNPs include neutral phospholipids, cholesterol, polyethylene glycol (PEG) lipids, and cationic ionizable lipids. Among them, cationic ionizable lipids, which are located on the surface of the LNPs, as positively charged ionizable amine groups, exhibit a cationic charge (at low pH) to interact with the anionic RNA during particle formation. During delivery, cationic ionizable lipids exhibit a net neutral surface charge at physiological pH. During internalization, cationic ionizable lipids exhibit a cationic charge at lower pH to facilitate membrane fusion and endosome formation.^156,157^ PEG-lipid provides LNP with a hydrophilic exterior, determining its thermodynamically stable size, and functions as a steric barrier to prevent aggregation during storage and to enhance LNP spatial stability.^158^ Cholesterol outside LNPs improves stability in vivo and intracellular delivery.^159^ Neutral phospholipids such as 1,2-distearoyl-sn-glycero-3 phosphocholine (DSPC) provide bilayer structural stability.^159^ In vitro, LNPs achieve high encapsulation efficiency of RNA vaccines.^160^ In vivo, LNPs protect RNA from degradation by enzymes from the endosome.^161^

The delivery processes of LNPs are dependent on endogenous pathways, such as the apolipoprotein E (ApoE)-low-density lipoprotein receptor (LDLR) pathway.^160–162^ LNP readily fuses with the target cell membrane. Then, LNPs are swallowed via TLR4-mediated endocytosis, forming the endosome in the cytoplasm.^163^ Finally, anionic lipids in endosomes bind to cationic lipids in LNPs to form a non-bilayer structure, which then unravels the endosomal membrane and releases the RNA vaccine (endosome escape)^164^ (Fig. 2).

The optimization of lipid components, LNP adjuvant and administration methods determine the distribution and expression kinetics of RNA vaccines. For lipid components, cationic ionizable lipids such as 1,2-dioleoyloxy-3-trimethylammonium propane chloride (DOTAP), 1,2-dio-leoyl-sn-glycerol-3-phosphoethanolamine (DOPE) and N-[1-(2,3-dioleoyloxy) propyl]-N, N,N-trimethylammonium chloride (DOTMA) are the primary substances for interacting negatively charged RNAs because they are positively charged in preparation buffer.^165–167^ In addition, lipids can further enhance the delivery efficacy by modifying their head and tail. For example, YSK12-C4 is a pH-sensitive cationic lipid, and its hydrophilic head and tail can improve endosomal escape.^168^ Moreover, adjuvant combined with LNP co-delivery is a potential strategy to enhance endosomal escape. For example, manganese (Mn) as delivery adjuvant can stimulate the STING pathway by directly activating synthesis of 2’ 3’-cyclic GMP-AMP (2’3’-cGAMP), which promotes the maturation of APCs and endosomal escape.^169^ For administration methods, local delivery is commonly realized by subcutaneous (SC) administration, intramuscular (IM) administration, and intradermal (ID) administration.^170^ Applicable administration can elicit locally strong and long-lasting humoral immune responses. For example, circRNA encoding the SARS-CoV-2 fusion RBD antigen has been encapsulated in LNPs. The formulated vaccine was intramuscularly injected into mice and rhesus macaques, resulting in increased levels of anti-SARS-CoV-2 RBD-specific IgG antibodies.^30^

**Fig. 6:**
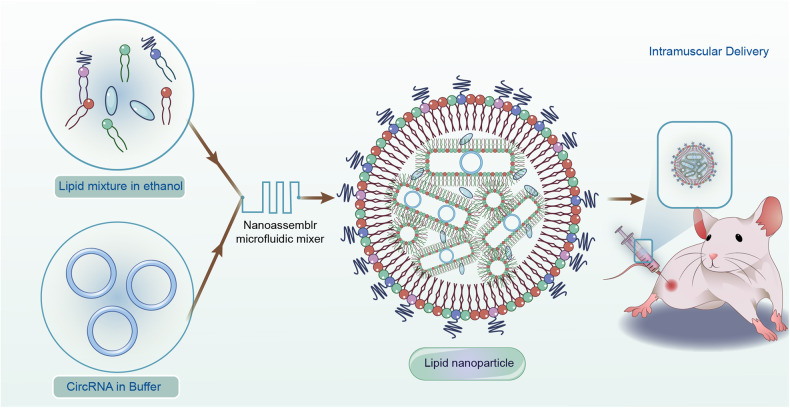
Schematic diagram of circRNA vaccine delivery. CircRNA was entrapped in lipid with microfluidic technology, and the obtained preparation were delivered into mouse through intramuscular injection

Recently, multiple novel cationic ionizable LNPs or strategies to deliver circRNA have been reported (Fig. 6). For example, charge-altering releasable transporters (CARTs) are temporarily cationic molecules capable of mediating mRNA delivery in mice.^171,172^ CARTs have been applied to the intravenous administration of circRNA with consistent circRNA-encoding capacity over 96 h.^69^ Moreover, multiarmed ionizable lipids, AX4-based lipid nanoparticles (AX4-LNPs), and commercially FDA-approved LNPs are used to efficiently package and deliver circRNA vaccines, exhibiting antigenic coding and immune responses when injected intramuscularly in vivo.^27–31^ Among them, AX4-LNP has the ability to be rapidly degraded in spleen cells, which can accelerate the release of circRNA vaccines.^28^ Besides, multiarmed ionizable lipids which is cooperated with a novel ionizable lipid is capable of inducing cytokine production. This LNP system is suitable for creating proinflammatory tumor microenvironment and activing cytotoxic T-cell activation without causing apparent side effects such as tissue damage.^29^ Collectively, the LNP delivery system is a promising platform for circRNA delivery. However, investigation is still in the early stage, and no preclinical trials have been performed. To date, a large number of cationic ionizable LNPs have been synthesized and tested as delivery platforms for nucleic acid preparations, and they are the most clinically state-of-the-art with the recent FDA approval of COVID-19-based modified mRNA vaccines.^25,173,174^ These novel strategies need further investigation for use in circRNA-based vaccines.

The better delivery systems are needed to bring circRNA to target cells. Typically, the reticuloendothelial system in the liver provides spaces large enough to allow the entry of these relatively large nanoparticles.^175^ The absence of a positive surface charge in plasma results in maximal delivery to hepatocytes and uptake by hepatocytes via ApoE-dependent, receptor-mediated endocytosis.^176^ Previously, LNPs administered intravenously or intramuscularly showed very strong vaccine accumulation in the liver, which triggered serious postvaccination liver injury.^177^ Thus, a delivery system capable of targeting or bypassing and rapidly metabolizing in the liver is needed. In this setting, many targeted delivery systems have been launched. For example, a study screened and optimized the structures of head amines, linker types, tail lengths, and tail combinations in LNP, formulating a novel LNP named 113-O12B that can perform targeted delivery of RNA to lymph node (LN) organs, which prevents hepatic damage and T-cell–dominant immune-mediated hepatitis.^178^ As the largest secondary lymphoid organ and host of a wide range of immunologic functions, the spleen is an irreplaceable terminus of vaccines. A novel LNP named YK009-LNP shows a favorable biodistribution pattern in the spleen and the primarily skeletal muscle site of injection, which contains abundant macrophages and other immune cells, exhibiting a faster elimination rate and low toxicity in the liver.^179^ Exceptionally, AX4-LNPs were further formulated with leading tail-branches, three coformulated excipient lipid molecules, and the incorporation of degradable linkers such as ester bonds into the aliphatic tails of lipidoids, forming a low-immunogenicity degradable LNP delivery system with a much faster elimination rate in the liver and spleen. This degradable LNP was successfully applied in vivo to deliver a circRNA vaccine and contribute to eliciting a robust immune response.^28^ Above all, this is beneficial for delivery studies of circRNA vaccines, and more clinical studies need to be conducted.

The delivery of agents into dendritic cells (DCs) is an alternative way to present antigens and control side effects. DCs express major histocompatibility complex (MHC) molecules that are used to bind antigens and then provide secondary signals and various cytokines for T-cell recruitment, activation and proliferation.^180,181^ Thus, DCs are an ideal vaccine target. Reportedly, LNP formulations can be decorated with ligands for their lectin and scavenger receptors, such as mannose, dectins and langerin, providing heightened specificity and internalization in DCs.^182^ Notably, human DC uptake of LNPs requires ApoE; thus, incubating circRNA-LNPs with exogenous ApoE or transfecting DCs with ApoE may improve targeting to DCs.^176^ In summary, targeting the manifestation of delivery systems to DCs not only avoids organ injury but also essentially promotes the adoptive immune response. However, the majority of targeted delivery systems can be efficiently used for in vitro delivery but have limited efficacy in vivo.^65^

### Other potential delivery strategies

Naked circRNA vaccines are achieved by directly injecting the circRNA solution without any delivery system. This strategy relies on APCs at the injection site and plays an adjuvant role in the treatment of solid tumors. APCs in the dermis, especially DCs, can take up an intradermal injection of naked mRNA, which is dependent on DC cell-mediated macropinocytosis. Then, antigens are expressed with the help of DCs.^183,184^ For agent injection, naked mRNA needs to be dissolved in solution, such as Ringer’s solution containing Ca^2^^+^, which facilitates RNA uptake.^185–187^ Hence, intradermal administration is a feasible route for the introduction of circRNA into APCs resident in the skin. Moreover, in clinical trials, ultrasound-guided percutaneous injection of the naked vaccine into inguinal lymph nodes can also trigger T-cell responses.^187^ This means that injecting circRNA into lymph nodes may easily achieve targeted delivery. Interestingly, a circRNA-based therapeutic platform for treating tumors could be achieved by direct intratumoral injection. Evidence has shown that in subcutaneous xenograft models of lung, melanoma, and colon cancer, direct intratumoral injection of a nondelivering circRNA vaccine with solutions such as Ringer’s solution can achieve localized cytokine expression, which modulates the local inflammatory microenvironment in tumor tissue and enhances tumor immunotherapy.^70^ In clinical studies, intratumoral injection of other vaccines in patients was determined to be safe and improved.^188–191^ Therefore, given that circRNA is injectable due to its inherent stability and lower immunogenicity compared to mRNA, more in vivo and clinical trials with naked circRNA vaccines need to be investigated.

Other delivery strategies used for mRNA delivery can be referenced for circRNA delivery. Since they are both RNA-based, circRNAs and mRNAs share similar encapsulation properties.^192^ Delivery systems currently used for mRNA vaccines also include polymetric nanoparticles, cationic nanoemulsions, exosomes, and so on. Polymetric nanoparticles, characterized by chemical/thermal stabilities, noncorrosiveness, suitable transition temperatures, high payload encapsulation efficiency, moderate protection ability, and customizable practicality, are potentially valuable for circRNA delivery.^193,194^ Cationic nanoemulsions in which cationic lipids are added to the formulation by electrostatic interactions were proposed as an effective RNA-based vaccine delivery system for the treatment of various diseases.^195,196^ Importantly, exosomes are extracellular nanometer-sized vesicles released by endogenous cells. These vesicles can serve as nanocarriers, possessing great potential to overcome some obstacles encountered in circRNA delivery due to their natural affinity to recipient cells and the inherent capability to shuttle the circRNA between cells.^197^ Exosome-mediated mRNA delivery for COVID-19 vaccination has been recently reported.^198^ Reactive astrocyte-derived exosomes were used to deliver mRNA vaccines to inhibit temozolomide resistance in glioma cells.^199^

With circRNA vaccine development, next-generation delivery systems are indispensable. Although currently available LNPs to deliver circRNA are promising, they have plenty of limitations, such as lower biocompatibility requiring organic solvents, complex production methods, and restrained injection routes, and are difficult to perform on large scales. More importantly, LNPs induce the activation of innate immune responses, as the ectogenic positive charges close to the cell surface are likely to be recognized as a signal of danger and contribute to triggering cascades of PRRs.^200^ Given these points, in the future, the development of circRNA vaccines may be disappointing with many early vaccines that may show promise in preclinical models but fail to translate into efficacy in the clinic. Solid lipid nanoparticles and nanostructured lipid carriers exhibit the fastest growth and have been developed to address some shortcomings of LNPs. Solid lipid nanoparticles consist of lipids and stabilizing agents such as surfactants and other coating materials, which are more stable than LNPs but may expel the incorporated drugs during long-term storage.^201,202^ On the basis of solid lipid nanoparticles, nanostructured lipid carriers are designed by introducing a small amount of lipid liquid.^203^ Nanostructured lipid carriers have a lower degree of crystallinity of the lipid core, higher drug-loading capacity, favorable physicochemical properties, long-term colloidal stability, enhanced bioavailability of nucleic acid agents, and improved agent release ability and can be taken orally.^204,205^ In addition, to maximize the therapeutic potential of circRNA vaccines, it is important to reduce off-target expression in nontarget cells and tissues. Novel delivery systems must be studied to realize the desired circRNA vaccine application.

## Potential applications of circRNA vaccines in disease

With the development of circRNA over the decades, its therapeutic applications for diseases have rarely been recognized. In 1990, IVT linear RNA was directly injected into mouse muscles for the first time, and the corresponding protein products were detected, which proved the feasibility of in vivo expression of artificially translated RNA.^206^ The first RNA-based vaccine candidate developed in 1993 was proven to induce both humoral and cellular immunity in vivo but exhibited unfavorable safety profiles and was susceptible to immune recognition and degradation by RNases.^207,208^ As a result, there was no breakthrough then in the research and application of RNA-based vaccines. In 2019, the outbreak of COVID-19 spread quickly and overwhelmed the local healthcare system of the epicenter. This pandemic led to a revolution in RNA-based vaccine technology. The mRNA vaccines against COVID‐19 were the first successful introduction of the entire RNA-based therapeutic platform.^209–211^ Due to the continuous encoding of antigens and the triggering of a long-lasting immune response compared to traditional vaccines, RNA-based vaccine platforms have been rapidly developed in various areas of biomedicine.

**Fig. 7:**
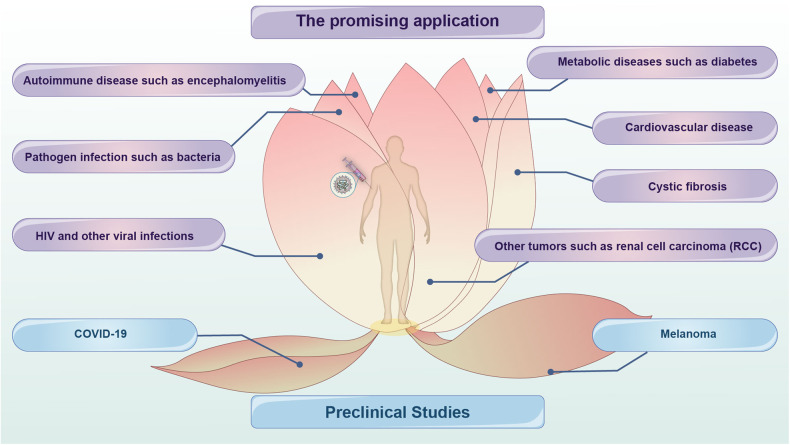
Potential application of circRNA vaccines. circRNA vaccines have yielded inspiring therapeutic and preventive outcome against COVID-19 and melanoma. Unfortunately, the application investigations of circRNA vaccines are at an early stage. In particular, the engineering circRNA as next-generation of RNA-based technologies shares the same ORF-dependent antigen expression technologies with mRNA vaccine. Attributed to higher stability, lower immunogenicity, and superior antigen-encoding capacity, circRNA can be re-engineered and replace the mRNA vaccine backbone for better therapeutic or prophylactic efficacy. Therefore, circRNA vaccines have promising applications in other viral infections, other tumors, autoimmune diseases, metabolic diseases, cardiovascular disease, and cystic fibrosis, etc

As a next-generation RNA-based vaccine platform, circRNA compensates for the shortcomings of mRNA and has the potential to become a vaccine candidate. However, the development of circRNA vaccines is still at a nascent stage, with very few reports on their in vivo studies and no relative clinical trials.^27–31^ More broadly, circRNA and linear mRNA vaccines share the same ORF-dependent antigen expression technologies. CircRNAs, as an upgraded platform, are expected to become a better vaccine therapy for various refractory diseases, including viral infectious diseases, tumors, pathogenic bacterial infections, autoimmune diseases, metabolic diseases, and other diseases (Fig. 7). In this section, existing circRNA vaccine studies will be discussed in the context of retrospective reviews of related diseases.

### Viral infectious diseases

Viral infections occur when viruses invade the body and replicate in target cells, leading to an inflammatory host response to viral infection and resulting in target organ tissue damage and dysfunction.^212^ The current countermeasures in preventing and treating viral infection, such as SARS-CoV-2 virus, influenza virus, human immunodeficiency virus (HIV), herpes simplex virus (HSV), respiratory syncytial virus (RSV), and varicella-zoster virus (VZV), have limited efficacy, despite the existence of antiviral drugs. These are probably attributable to its high infectious capacity and its marked immunogenicity and mutagenic plasticity.^213,214^ Vaccines against viruses that are licensed for use in humans are prophylactic vaccines that prevent viral infection via the induction of virus-neutralizing antibodies.^14^ Considering their protective effectiveness, economy of manufacture, and the possibility of producing antigens in vitro, researchers have turned their attention to new vaccine technologies. At present, RNA-based vaccine technology has been demonstrated to stimulate the production of antibodies against a virus in humans with good tolerability.^215^

COVID-19 has emerged as a global health threat due to its accelerated geographic spread over the last 3 years. Today, COVID-19 is still a latent threat to human health. In the majority of cases (79.5%), the patients who died with COVID-19 were suffering from underlying diseases.^216^ Old people aged >65 years are more vulnerable to developing severe and critically severe infections than other age groups.^217^ Hence, more adaptive therapeutic or prophylactic methods are needed to address the emergence of the evolving SARS-CoV-2 virus and its variants.

The SARS-CoV-2 genome consists of 15 ORFs encoding 29 proteins, of which the spike protein is thought to be the receptor for viral binding to the host cell.^217,218^ Virus entry into host cells occurs by binding to its receptor, angiotensin-converting enzyme 2 (ACE2), through the RBD at the C-terminus of the S1 subunit in the spike protein.^210,219^ The Omicron variant carries more than 30 mutations in the spike protein, 15 of which are located in the RBD.^220^ Therefore, the RBD has emerged as an antigen in the therapeutic strategy to design vaccines. Based on current studies, multimerized antigens are better at engaging interactions with B-cell receptors, thereby facilitating the generation of high-affinity antibodies compared to monomeric antigens.^221^ Thus, strategies have been developed to enhance RBD protein immunogenicity and to improve antibody titers, such as construction of dimers, trimers or polymers, antigen mutation, fusion of antigen, and formulation of wild-type and prefusion stable antigens.^222–225^

Recently, four studies have reported on circRNA vaccines to treat SARS-CoV-2 and its emergent variants. Among them, three use the group I intron-mediated PIE system for circRNA synthesis. The SARS-CoV-2 circRNA vaccine (VFLIP-X) encodes a VFLIP-X antigen, an RBD mutation that comprises emerging variants, including K417N, L452R, T478K, E484K, and N501Y, and the comutation at residue 614 (D614G).^31^ The SARS-CoV-2 circRNA vaccine (fused RBD antigen) encodes a fused RBD antigen, the N- and C-termini of which were fused with human tissue plasminogen activator (tPA) and trimerization motif (foldon) of bacteriophage T4 fibritin protein, respectively, to enhance the secretion of the antigen and improve its immunogenicity.^30^ The SARS-CoV-2 circRNA vaccine (delta RBD antigen) encodes the RBD on the spike glycoprotein of the B.1.617.2 variant.^28^ In addition, a SARS-CoV-2 circRNA vaccine (RBD dimer) was synthesized by the group II intron-mediated PIE system, which encodes a fused RBD dimer antigen in which the RBD dimer was fused with the foldon from T4 fibritin.^27^

SARS-CoV-2 circRNA vaccines could confer durable antigen-encoding efficiency and high stability. CircRNA without any modifications exhibits high stability and RNase resistance, mainly attributed to its circular conformation without 5’ caps or 3’ poly(A) tails.^226^ Owing to this property, endogenous circRNAs have a markedly longer half-life than their full-length linear RNA counterparts.^227^ Moreover, the coding longevity of artificially translatable circRNAs is much longer than that of the corresponding nucleoside-modified linear mRNA. The circRNA vaccine (fused RBD antigen) can be stored at 4 °C, 25 °C, or 37 °C for up to 28 days. For antigen expression, the circRNA vaccine (fused RBD antigen) was transfected into human and murine cells, and abundant RBD antigens were detected in the supernatant.^35^ Moreover, the circRNA vaccine produced higher levels of RBD antigens than the corresponding mRNA vaccine.^30,31^ In addition, the biodegradable LNP-encapsulated SARS-CoV-2 circRNA vaccine (RBD dimer) exhibits high stability and can be stored for up to six months at 4 °C or during six cycles of freezing and thawing.^27^

The SARS-CoV-2 circRNA vaccine could confer higher proportions of neutralizing antibodies. Theoretically, circRNA vaccines are capable of longevity, thus prolonging the production of antigens and thus antigen presentation, which may induce persistent humoral immunity. The circRNA vaccine (VFLIP-X) uses the LNP delivery system and is immunized by intramuscular injection with 5 μg of circRNAs, which induce a strong neutralizing antibody response against various SARS-CoV-2 variants in mice.^31^ CircRNA vaccine (fused RBD antigen) is encapsulated with LNP and is immunized by intramuscular injection with 10 μg of circRNA at a 2-week interval in mice, which elicited a higher proportion of Th1-biased responses, RBD-specific CD8^+^ T-cell responses and consistently higher ratios of neutralizing/binding antibodies.^30^ The SARS-CoV-2 circRNA vaccine (delta RBD trimer antigen) is encapsulated by a novel degradable LNP delivery system and is administered by intramuscular injection with 30 μg of circRNA in mice, which induces strong RBD/spike-specific antibodies in serum and significantly increase specific CD4^+^ and CD8^+^ effector memory T cells without any systemic adverse events.^28^ LNP-encapsulated SARS-CoV-2 circRNA vaccine (RBD dimer) (20 μg) can induce a strong RBD-specific memory B-cell response and RBD antibody in mouse serum.^27^

SARS-CoV-2 circRNA vaccines exhibit broad-spectrum protection against various SARS-CoV-2 variants and prevent severe symptoms of COVID-19. The VFLIP-X circRNA vaccine and the fused RBD antigen circRNA vaccine both inhibit various SARS-CoV-2 variant infections in mice, including B.1.1.7, B.1.351, B.1.617.2, and B.1.1.529. In addition, rhesus macaques were intramuscularly injected with 100 or 500 μg of circRNA vaccine (fused RBD antigen), which induced high levels of specific IgG and Th1-biased responses in the serum collected after 2 weeks. Further viral load and histopathological assays indicated that the circRNA vaccine effectively reduced viral genomic RNA burden and protected the lungs of rhesus macaques from the severe symptoms of COVID-19, such as local pulmonary septal thickening, moderate hemorrhage in the pulmonary septum, the number of scattered dust cells, and massive inflammatory cell infiltration.^30,31^

In the face of the normalization of the COVID-19 pandemic, countermeasures have shifted from treatment to prevention. People with underlying diseases, especially aged individuals, are susceptible to serious SARS-CoV-2 infection. There is still a need for SARS-CoV-2 vaccines. The development and clinical trials of SARS-CoV-2 mRNA vaccines have been accelerated. However, the research process and clinical application of mRNA vaccines have been hampered, in part, by their instability, high immunogenicity, high storage costs and inability to be produced on a large scale. As a next-generation RNA-based vaccine platform, circRNAs compensate for most of the disadvantages of mRNAs. SARS-CoV-2 circRNA vaccines require further in vivo studies to advance to clinical trials and therapeutic applications.

CircRNA vaccines also have potential applications in other viral infectious diseases. RNA-based vaccines have been established against some viruses. For example, an mRNA vaccine encoding a combination antigen of full-length matrix-2 ion channel, hemagglutinin stalk domain, influenza virus membrane-bound neuraminidase and wild-type nucleoprotein has been developed to protect against influenza virus infection.^228^ An mRNA vaccine encoding a combination antigen of HIV-1 Gag, Pol, and Nef proteins has been developed to protect against HIV infection.^229^ An mRNA vaccine encoding different forms of the RSV F protein has been developed to protect against RSV infection.^230^ An mRNA vaccine encoding three HSV-2 glycoproteins, gC2, gD2, and gE2, has been developed to prevent HSV-induced genital herpes.^231^ An mRNA vaccine encoding varicella-zoster virus (VZV) gE antigen has been developed to protect against VZV infection.^232^ An mRNA vaccine encoding rabies glycoprotein prevents rabies virus infection and has shown good tolerance in clinical trials.^233^ An mRNA vaccine encoding a combination antigen of glycoprotein B and pentameric complex has been developed to protect against human cytomegalovirus in macaques with preexisting immunity.^234^ Moreover, mRNA vaccines have been reported to have excellent results in preventing infection by ectromelia virus, dengue virus, and hendra virus.^235–237^ As noted above, based on the application prospects of RNA vaccine technology, circRNA will certainly become a better virus treatment option. Alternatively, circRNA can be adaptably used to encode the mentioned antigens, producing superior efficacy compared to mRNA vaccines. In future trials, circRNAs may show superior efficacy, which warrants further investigation.

Interestingly, circRNAs can directly encode antibodies to prevent viral infections. Neutralizing antibodies are proteins with immune effects produced by B cells when stimulated by antigens, mainly in blood and tissues.^238,239^ These antibodies specifically recognize viruses, bacteria, and toxins and bind them through long parts of the variable chains, exerting effectual effects in preventing and treating SARS-CoV-2 viruses, HIV or tumors in animal models and humans.^240–247^ RNA-based technology also assembled the strategy, with delightful therapeutic effects for chikungunya virus, toxins, HIV, malignant tumors, and SARS-CoV-2 virus.^248–252^ In particular, a circRNA-based vaccine was constructed to encode SARS-CoV-2-specific neutralizing antibodies, which effectively neutralized the SARS-CoV-2 pseudovirus.^30^ This means that circRNA has the potential to become a neutralizing antibody-encoding platform.

### Tumors

Tumor immunotherapy aims to activate host antitumor immunity to create a tumor-suppressive microenvironment, ultimately achieving tumor elimination and improving overall patient survival. Immune checkpoint blockade (ICB) and chimeric antigen receptor T cells (CAR-T cells) are commonly used tumor immunotherapy strategies.^253^ Recently, tumor vaccines have become a promising strategy for antitumor immunotherapy. Vaccines expressing tumor-associated antigens (TAAs) or tumor-specific antigens (TSAs) can stimulate inflammatory induction, specifically recruit immune cells and destroy tumor cells that express high levels of these antigens, ultimately achieving sustained tumor killing through immune memory. Thus, cancer vaccines could theoretically provide specific, safe and well-tolerated therapeutic effects compared to other types of immunotherapy. To date, there is only one available report of an in vivo study of circRNA vaccines for the treatment and prevention of tumors, and it was concerning melanoma.

The incidence of malignant melanoma has been increasing worldwide since the start of the 21st century. As one of the most frequent cancers in fair-skinned populations, melanoma mostly affects young and middle-aged people, resulting in an important socioeconomic problem.^254^ Moreover, males are ~1.5 times more likely to develop melanoma than females.^255^ Systemic therapies for melanoma have been dramatically revolutionized by the development of targeted therapies, such as BRAF and MEK inhibitors, and immunotherapies, such as anti-PD-1 antibodies. However, these innovative agents are still restricted in treating patients with advanced melanoma.^256^ RNA-based cancer vaccine platforms have been developed with encouraging results in terms of their capacity to activate the melanoma immunity cycle and preserve the body’s safety.^187,257^

Based on studies related to melanoma antigens, there are four classes of antigens that can be used to design melanoma circRNA vaccines. First, shared antigens used in melanoma vaccines enable broad application. Tyrosinase, TRP-2, chicken ovalbumin (OVA), and gp-100 are common source proteins that are also expressed on normal melanocytes and on a few other pigmented cells.^258^ Second, mutated antigens are raised through tumorigenesis, and they are absent in normal cells. BRAF, KIT, and NRAS mutations are common mutated antigens in melanoma.^259,260^ Third, germline antigens are expressed in the placenta or testis as immune privilege markers.^261^ MAGE-A1, MAGE-A3, BAGE, GAGE, and NY-ESO-1 are cancer germline antigens that have been identified.^262,263^ Fourth, the term neoantigen refers to newly expressed or acquired antigens, as in genomic mutations found within tumors but not in normal somatic cells. Because of the unique genetic sequence, a neoantigen may avoid preexisting central tolerance that is expected with shared antigens and reduce the risks of off-target autoimmune reactivity. Therapeutic T cells may respond more strongly to mutated neoantigens than to shared antigens.^264^ However, a disadvantage of neoantigens is that they require the synthesis of personalized vaccines. These mutated epitopes can be engineered into vaccines using a personalized approach.^187^ In addition, MHC class I and II molecules in APCs can specifically present phosphorylated antigens, suggesting that phosphorylated antigens have higher immunogenicity.^265,266^

Recently, one study was reported on circRNA-based vaccines to treat melanoma. This circRNA vaccine was synthesized in vitro using the group I intron-mediated PIE system and initiated translation using CVB3 IRES elements. The shared antigen OVA (257-264; SIINFEKL) was used to induce an immune response. This circRNA vaccine has a higher half-life than that of the corresponding nucleoside-modified linear RNA. Notably, this circRNA is encapsulated with LNP and is immunized by intramuscular injection with 10 μg of circRNA at a 1-week interval in mice, exhibiting superior efficacy in treating the “immune-excluded” MC38 tumor model, “immune-desert” B16 orthotropic melanoma. This vaccine platform exhibited extraordinary antimetastatic performance in a B16 lung metastasis model.^29^

Melanoma circRNA vaccine (OVA) activates the cytotoxic T-cell-mediated tumor-killing response. Unlike prophylactic vaccines that provoke antibody-based B-cell responses and antibody-mediated immunity responses, cancer RNA vaccines should induce a robust cytotoxic T-cell response.^267^ This melanoma circRNA vaccine induced sustained antigen-specific cytotoxic T-cell responses, and no apparent toxic side effects were observed in the main organs.^29^ Owing to its efficient encoding ability allowing prolonged antigen presentation, artificial circRNA is better suited to continuously active tumor-killing T-cell function. It’s worth noting that cytotoxic T cells work in a proinflammatory immune microenvironment. The circRNAs are less immunogenic and do not provide a proinflammatory microenvironment. This melanoma circRNA vaccine used a novel ionizable lipid cooperated with the LNP delivery system to induce a proinflammatory immune environment suitable for cytotoxic T-cell activation.^29^

Melanoma circRNA vaccine (OVA) has prophylactic effects. As vaccines, circRNA vaccines should achieve prophylactic effects against cancers to protect susceptible people. To detect the prophylactic effects, mice were injected intramuscularly with 10 μg of melanoma circRNA vaccine (OVA) at 1-week intervals. Significant SIINFEKL-MHC-I tetramer-positive cytotoxic T cells were detected in the peripheral blood on Day 14 after 2 doses of injection. This circRNA vaccine protects mice from survival for 2 months post tumor cell injection.^29^

In summary, the melanoma circRNA vaccine (OVA) can provide immune protection for melanoma induction, exert more efficient therapeutic effects on patients with melanoma, and prevent lung metastasis. However, the potential role in other tumors is unclear. Recently, mRNA vaccines for renal cell carcinoma (RCC), glioblastoma, and acute myeloid leukemia (AML) have demonstrated an active immunotherapy response.^268–270^ We propose to explore the role of circRNA vaccines in these and other malignancies.

CircRNA can provide an alternative therapeutic option for tumor treatment. In terms of tumor treatment from mRNA vaccines, it is more effective at stimulating the functions of APCs.^271^ In detail, DC-based RNA vaccine technology is promising in the treatment of various tumors. This therapeutic option delivers RNA molecules to DCs to stimulate adoptive immunity and induce cancer immunotherapy.^257^ The activation of DCs elicits an inflammatory tumor microenvironment, which has an extremely strong effect on immune cell infiltration. DC-based mRNA vaccine technology has demonstrated superior therapeutic effects in glioblastoma, acute myeloid leukemia, and renal cell carcinoma.^269,272,273^ However, vaccine therapy for tumors is progressing slowly, which may be related to the heterogeneity of tumor cells and the susceptibility of antigens to mutation. Future research on the selection of highly immunogenic antigens and their introduction into circRNA-based vaccine technology will greatly enhance the effectiveness of tumor immunotherapy.

It is worth mentioning that antigens are often unfavorable for T-cell activation.^274,275^ Many TAAs trigger clinically ineffective T-cell responses, as observed in many vaccine trials, and T-cell tolerance may also contribute to the weak immune response.^276,277^ To address this limitation, in addition to formulating the antigens, incorporating vaccine adjuvants, such as interferons, TNF, IL-12, IL-2, and IL-6, can promote the maturation of APCs, thus fulfilling the function of APCs in enhancing antigen presentation and immune responses.^275,278,279^ Moreover, adjuvants stimulate PRRs on APCs. These PRRs create a proinflammatory microenvironment that affects T-cell activation and proliferation.^280^ In addition, some recombinant cytokines, such as type I IFN, have direct antiangiogenic effects and induce tumor cell apoptosis in melanoma.^281,282^ Therefore, adjuvant-type vaccines were first implemented in mRNA-based technology, which encodes various cytokines and confers antitumor immunity, as well as in combination with antigen-type vaccines to improve therapeutic effects.^283,284^ Despite the potential danger of cytokine storms, using RNA-based vaccines to deliver to targeted cells and to induce local cytokine encoding can produce appreciable therapeutic effects, and the combination of other therapeutic options will greatly enhance therapeutic effects.^285,286^

CircRNAs are worthy of research and clinical trials as adjuvant-based vaccines. Recently, a novel circRNA vaccine was established to encode a combination of cytokines (IL-15, IL-12, GM-CSF, and IFN-a, 2b), which achieved successful modulation of intratumoral and systematic antitumor immune responses. This vaccine resulted in marked suppression of tumor growth in the syngeneic mouse colon and melanoma tumor model.^70^ This vaccine can further facilitate anti-PD-1-mediated immune therapy.^70^ Therefore, adjuvant-type circRNA vaccines encoding cytokine mixtures can elicit a notable tumor-suppressive effect by boosting T cells to facilitate immune therapy. More in vivo studies, especially clinical trials, are necessary.

### Other diseases

Considerably more work will need to be done to determine the therapeutic roles of circRNA vaccines and circRNA-based technologies in other diseases. Pathogens such as bacteria, parasites, and fungi lead to serious public health problems. In particular, resistant bacterial infections always increase the risk of poor clinical outcomes and acute death, as they consume more medicines and medical resources than other infections.^287^ Therefore, the development of relevant vaccines is of great significance for early treatment and prophylaxis against bacterial infections. Antibacterial mRNA vaccines are ideal tools to protect mice from infection by group A (GAS) and group B (GBS) streptococci.^288^ However, this type of vaccine is still under preclinical evaluation. In addition, accumulating evidence suggests that RNA-based therapeutic platforms have shown satisfactory efficacy in other diseases, including autoimmune diseases, such as encephalomyelitis, and metabolic diseases, such as type 2 diabetes, cardiovascular disease and cystic fibrosis, with safety and reactogenicity profiles.^289–294^ While studies did not confirm the effectiveness of circRNA, they did partially substantiate that RNA-based therapeutic platforms could be practical for the treatment or prevention of such diseases, which certainly adds to our understanding of the potential application of circRNA. It is worth noting that circRNA may adapt to antigenic sequences on mRNA, which could be a major advantage for the future development of circRNA vaccines. However, current laboratory experiments on artificial circRNA encompass design optimization and disease feasibility research, and no available clinical efforts are found. Due to the early stage of circRNA vaccines, researchers are moving toward the fields of antiviruses and antitumors. Expanding the boundaries of experimentation to accelerate clinical research would make this a fruitful area.

### Potential applications of artificial circRNA-based therapeutic platforms

In addition to utility as vaccines, circRNA-based technologies have broader therapeutic prospects. The miRNAs are highly expressed in various pathological processes.^295–299^ The inhibition of miRNAs by natural circRNAs relieves the excessive repression of their target mRNAs and restores the normal cell phenotype, which is regarded as a promising therapeutic strategy. Because natural circRNAs act as sponges that can soak up miRNAs, titrate miRNA activity and derepress mRNAs, many techniques have been developed to decipher the complex circRNA sponge network and to reverse pathological conditions.^300–304^ Although these methods do not overturn the role of miRNA, engineering sponge-type circRNAs may have important prospects in the treatment or prevention of diseases.

CircRNAs synthesized in vitro with engineered sequences have been used to regulate miRNA activity. For example, artificial circRNA, which contains multiple miRNA binding sites, can be delivered into targeted cells to function as ceRNAs for miR-122, miR-212, and miR-132, reducing the life cycle of hepatitis C virus (HCV) and attenuating pressure overload-induced cardiac hypertrophy.^73,305^ In addition, circRNA-based techniques can also be applied to regulate protein sponge function. For example, artificial circRNAs with CA-repeat or CA-rich sequence clusters can efficiently and specifically modulate splicing processes by acting as a sponge to affect the translocation of heterogeneous nuclear ribonucleoprotein L (hnRNPL) between the nucleus and cytoplasm.^74^ This has promising applications in hnRNPL-mediated genetic diseases. Therefore, by engineering the sponge-type circRNA platform, the equilibrium between circRNAs and other interacting molecules can be disrupted to exhibit disease therapeutic or regulatory gene expression effects. However, the duration and therapeutic significance of this platform is unclear, and there is blindness in prophylactic treatment. However, this is an important type of circRNA in the application of therapeutic diseases, as there is a large amount of sponge behavior in vivo.

For the design of sponge-type circRNAs, the introduction and optimization of homologous miRNA binding sites (MBSs) in linear RNA precursors is essential for intact circRNAs to sequester miRNAs. In conventional design, linear RNA precursors must contain an array of MBSs to competitively and specifically neutralize miRNA with reverse complementarity, which allows circRNA to block a whole family of related miRNAs.^306^ MBSs are designed to match the mature sequences of targeted miRNAs, which can be identified by using databases such as miRBase, circBank, circNet, miRTarBase, CircInteractome, starBase v2.0 and regRNA 2.0.^307–313^ For the assembly strategy, the MBS elements in linear RNA precursors should contain mismatches rather than perfect antisense sequences, as perfectly base-paired sequences can form more stable interactions with miRNA and would be vulnerable to Ago2-mediated endonucleolytic cleavage.^314,315^ Much evidence shows that sponges containing interspersed mismatch sites misaligned with miRNAs are more effective in matching miRNAs.^73,316,317^ These mismatches can be designed to delete 1 or 3 bases from the MBSs, forming the bulge sites.^317^ Furthermore, the number of MBSs should be less than 12 because increasing the number may result in diminishing marginal utility.^316^ In addition, the insertion of spacers, which are scrambled sequences 12 bases in size, can synergistically reduce the risk of binding unintended intermediaries.^73,318^ Ultimately, the RNAhybrid tool can be used to verify the binding efficiency of designed circRNA sponge sequences to target miRNA sequences.^319,320^

In addition, in vitro-synthesized endosomal circRNA mimics can also be used to treat diseases. Given the protein encoding ability of artificial circRNA-based technology, circRNAs can serve as transcripts for the expression of tumor or disease suppressors. This role of circRNA suggests that in vitro synthetic circRNA can be engineered to mimic endogenous translatable circRNA. To date, several studies have reported that natural circRNA-encoding peptides can control disease occurrence and development. For example, a natural circRNA is capable of encoding an 87 amino acid peptide called PINT87aa. This PINT87aa can inhibit the tumorigenesis of glioblastomas.^3^ Circ-SHPRH, as a natural circRNA, is known to encode an unusual protein known as SHPRH-146aa. SHPRH-146aa is involved in the onset of neurodegenerative diseases.^321^ CircZKSCAN1 encodes circZKSaa, which promotes the ubiquitination of mammalian target of rapamycin (mTOR) to suppress hepatocellular carcinoma (HCC) development.^11^ However, artificial circRNA is not currently being used to mimic endogenous translatable circRNA. This field will receive increasing attention and development due to the maturation of current in vitro circRNA synthesis technology and the improvement of protein compilation capability.

## Conclusion and prospects

Due to their stability, long life, low immunogenicity, and translatability, engineering circRNAs has become a new topic of high interest in the field of vaccine research. In general, five pivotal challenges require attention: designing circRNAs with low immunogenicity and high antigenic yields, improving the circularization efficiency of linear RNA precursors, adequately purifying to avoid contaminants, establishing suitable delivery systems and enabling disease therapeutic applications (Fig. 2). More technology and experimental methods, as well as in vivo experiments and clinical trials, need to be improved and developed.

Based on the current findings, there are a few suggestions that may serve as directions for future research:

First, nucleotide modifications may synergistically enhance circRNA vaccine stability and reduce immunogenicity. Previous studies imply that mRNA vaccines require chemical nucleoside modifications to achieve stability and avoid activation of the innate immune system.^23^ For example, the incorporation of nucleotide modifications, including 5-methylcytosine (m5C), m6A, 5-methyluridine (m5U), 2-thiouridine (s2U), and N1-methylpseudouridine (m1Ψ), or optimization of free-ending poly(A) and 3’ UTR into linear precursor molecules abrogates the immune response by evading the activation of TLR-3, -7, and -8.^36,322–324^ In addition, substitution with m1Ψ, m6A, and s2U in mRNA molecules suppresses the degradation of RNA by RNase L.^325^ Moreover, unmodified mRNA can induce the activation of RNA-dependent protein kinase (PKR).^37^ PKR is one of four kinases known to phosphorylate the subunit of translation initiation factor 2 (eIF2) and repress translation.^326,327^ Hence, the requisite modification can enhance RNA vaccine stability and prevent the innate immune response. Indeed, evidence demonstrates that unmodified circRNA activates RIG-I and innate immune signaling, but the m6A modification in circRNAs apparently inhibits innate immune responses.^68^ It appears that, given the most abundant modification on the RNA polyA tail in mammals, m6A modification tags and signals exogenous circRNA as endogenetic to escape the surveillance of innate immunity, while foreign circRNAs are unmodified and trigger innate immune responses.^99–101^ Of note, circRNAs without exogenous sequences and with sufficient purification have minimized immunogenicity.^38,123^ Under these conditions, m6A modification is not necessary to reduce the immunogenicity of circRNA. In addition, circRNA containing 5% m6A was found to be more resistant to nucleases and showed moderate resistance to degradation in FBS solution.^69^ Nonetheless, human heat-responsive protein 12 (HRSP12) functions as an adapter to bridge YTHDF2 and RNase-P/MRP, and RNase MRP are essential ribonucleoprotein complexes that function as endoribonuclease.^328,329^ For this reason, m6A modification may be involved in endoribonucleolytic cleavage of circRNA in the cytoplasm, indicating that m6A modification may affect the lifespan of circRNA and further evidences are needed to clarify the role of m6A in circRNA stability in vivo. Noteworthy, other nucleotide modification types have also been suggested to affect the immunogenicity and function of circRNA.^68^ But, unconventionally, m1Ψ, the most commonly used nucleoside modification, favors mRNA vaccines, while circRNA does not benefit from m1Ψ modification in terms of protein expression and immunogenicity.^38^ This difference is just the tip of the iceberg, and more research is still needed. In summary, the incorporation of nucleotide modifications or optimal natural sequences may provide multiple benefits, enabling circRNA to serve as a versatile, flexible, and safe means for vaccine therapies.

Second, circularization methods need to be further investigated. The circularization efficiency of linear precursors decreases with their length, which is one of the limitations of circRNA vaccines. In this context, as the most commonly used ribozyme ligation approach, the PIE method has merits in performing ligation of larger linear RNA, and the reaction is relatively simple. However, the group I intron-based PIE method unavoidably generates undesired and indistinguishable contaminants, which ultimately lead to purification difficulties. Of note, synthesized circRNA via PIE methods tend to form stable intramolecular duplexes, which are basically due to the residual exogenous sequences. These RNA duplexes as residual nonnatural or nonfunctional elements are potential immanent initiators of the innate immune response, resulting in side immunogenicity, albeit after thorough purification. And these RNA duplexes form complicated three-dimensional structures and distort the folding status of original circRNAs and are likely to affect their normal function.^123^ Accordingly, improvement of circularization methods may be the primary means to reduce the immunogenicity of circRNA, and alternative circularization methods are needed. For instance, the Clean-PIE system ingeniously identifies the optimal circularization site by screening the protein-coding region or IRES region and achieves high circularization efficiency (>90%) with no exogenous sequence introduction and precise design sequence. The circularization products synthesized by this system exhibit lower immunogenicity and higher protein expression.^330^ However, this approach is limited to different ORFs and IRESs. Furthermore, circRNA made by T4 RNA ligases and chemical approaches without extraneous fragments exhibits minimal byproducts.^117^ And synthesized circRNA by T4 RNA ligase exhibit lowest immunogenicity.^123^ However, chemical and enzymatic strategies normally show reduced yields for long linear RNA precursors.^124^ Optimizing synthetic methods to produce larger circRNAs are necessary. In addition, the group II intron-based PIE method is capable of generating circRNAs without the introduction of any extrinsic sequence.^27,129^ Therefore, there is a fundamental need for the further investigation of scalable and improved circularization methods to produce products containing only functional sequences and more efficient subsequent preparation.

Third, new purification methods to obtain high-purity circRNA are urgently needed. Inadequate purification will retain some contaminants from synthetic reactions that are highly immunogenic. These contaminants are not only the source of side effects, but also the culprits that circRNAs are degraded in cells. Indeed, the highly purified circRNA vaccine can further reduce side immunogenicity and promote protein expression.^35^ Recently, orthogonal methods have been identified to confirm circRNA purification. These methods include several additional analyses to verify the major product of in vitro-synthesized circRNA, including gel electrophoresis, RNase R digestion, oligonucleotide-guided RNase H digestion, and HPLC.^139^ Among them, HPLC has superior resolving power and has been used in vaccine characterization. HPLC is a simple, fully automated, well-documented, and highly popular method. It is much easier to build a harmonized protocol, an international standard or large-scale industrial manufacturing. However, the complete elimination of RNA contaminants from the synthesis reaction, especially the PIE reaction, is still not achieved, even after HPLC.^35^ The explanation is that due to RNase R treatment is not sufficient to degrade all linear RNA, the rudimental noncircular components with triphosphorylated tails are stronger immunogenic. And HPLC fails to remove linear RNA with similar molecular weight. Despite the addition of phosphatase treatment after HPLC purification and before RNase R digestion can dramatically reduce the immunogenic, this strategy undoubtedly causes problems for post-protein elimination and high-throughput production.^38^ Therefore, the purification strategy is not yet mature, and the novel purification methods need to be improved and developed.

Finally, circRNA-based therapeutic platforms have more potential roles in disease treatment. Considering the identification of endogenous circRNAs and the elucidation of their functions, circRNA sponging miRNAs to alleviate the degradation or translation repression of target mRNAs has been increasingly described, especially in processes related to oncology.^331,332^ It is worth mentioning that the establishment of engineered circRNAs to mimic endogenous or exosomal noncoding circRNAs, which enables the manipulation of ceRNA interactions, may engender new research fields or disease therapeutic schedules. More broadly, many translatable natural endogenous circRNAs have been identified that have important roles in disease occurrence and progression.^1,6,11^ Artificial circRNA to express peptides for disease treatment may also confer medical benefits, even enabling genetic engineering and disease therapy. However, this therapeutic platform relies on the production of disease-suppressive proteins, which is very little in current reports. Notably, circRNA-based technologies are also emerging in other disease treatment strategies, including neutralizing antibody-encoding circRNA technologies and adjuvant-type circRNA technologies.^30,70^ These application prospects could further expand the medicinal value of engineered circRNAs. Noteworthy, due to circRNAs perform many biological functions, in vitro-synthesized circRNAs may cross-react with their latent interactions, resulting in unwanted functions or even serious side effects. Therefore, the sequences of artificial circRNA should be specific and are adequately tested for biosafety and efficacy in preclinical evaluations. Overall, artificial circRNAs have potential as a tool for molecular biology and medicine in the development of new strategies to combat human diseases.

In summary, there is an urgent need to develop and improve advanced methods for in vitro artificial circRNA vaccine design, synthesis, purification, delivery, and therapeutic application. Building on many of the earliest efforts, multiple approaches can be reciprocally beneficial. Future investigations should focus on methodological development, improvement, application, and clinical trials. By enabling the integrity of circRNA vaccines, novel circRNA-based technologies will pioneer new directions for disease treatment and prevention. We look forward to the successful clinical translation of circRNA vaccines.
